# Supplementary material for: Actionable druggable genome-wide Mendelian randomization identifies repurposing opportunities for COVID-19
Source: Nat Med. Author manuscript; Available in PMC 2022 Jul 5. (PMC7612986; doi:10.1038/s41591-021-01310-z)
Supplement: Supplementary Information [file EMS146273-supplement-Supplementary_Information.pdf]

2. Supplementary Information:

A. Flat Files

Complete the Inventory below for all additional textual information and any additional Supplementary Figures, which should be supplied in one combined PDF file.

- **Row 1:** A combined, flat PDF containing any Supplementary Text, Discussion, Notes, Additional Supplementary Figures, Supplementary Protocols, simple tables, and all associated legends. Only one such file is permitted.

- **Row 2:** Nature Research's Reporting Summary; if previously requested by the editor, please provide an updated Summary, fully completed, without any mark-ups or comments. **(Reporting Summaries are not required for all manuscripts.)**

**Item**	**Present?**	**Filename**<br>**This should be the name the file is saved as when it is uploaded to our system, and should include the file extension. The extension must be .pdf**	**A brief, numerical description of file contents.**<br>i.e.: ***Supplementary Figures 1-4, Supplementary Discussion, and Supplementary Tables 1-4.***
**Supplementary Information**	Yes	Supplementary_Information.pdf	Supplementary Methods and Supplementary Figures 1-2
**Reporting Summary**	Yes	nr-reporting-summary.pdf	

B. Additional Supplementary Files

Complete the Inventory below for all additional Supplementary Files that cannot be submitted as part of the Combined PDF.

- Do not list Supplementary Figures in this table (see section 2A)
- Where possible, include the title and description within the file itself

- Spreadsheet-based tables & data should be combined into a workbook with multiple tabs, not submitted as individual files.
- Compressed files are acceptable where necessary. ZIP files are preferred.
- Please note that the *ONLY* allowable types of additional Supplementary Files are:
    - Supplementary Tables
    - Supplementary Audio
    - Supplementary Videos
    - Supple
    - Supplementary Data, for example: raw NMR Data, Cryo-EM Data, Computational Data, Crystallo

**Type**	**Number** If there are multiple files of the same type this should be the numerical indicator. i.e. "1" for Video 1, "2" for Video 2, etc.	**Filename** This should be the name the file is saved as when it is uploaded to our system, and should include the file extension. i.e.: *Smith_ Supplementary_Video_1.mov*	**Legend or Descriptive Caption** Describe the contents of the file
Supplementary Table	1-16	supplementary_tables.xlsx	Supplementary Tables 1–16

Editor summary: Large-scale Mendelian randomization and colocalization analyses using gene expression and soluble protein data for 1,263 actionable druggable genes, which encode protein targets for approved drugs or drugs in clinical development, identify IFNAR2 and ACE2 as the most promising therapeutic targets for early management of COVID-19.

Editor recognition statement: Joao Monteiro was the primary editor on this article and managed its editorial process and peer review in collaboration with the rest of the editorial team.

Reviewer recognition statement: Nature Medicine thanks David Evans, Steven Wolinsky, and the other, anonymous, reviewers for their contribution to the peer review of this work.
